# Orai1α and Orai1β support calcium entry and mammosphere formation in breast cancer stem cells

**DOI:** 10.1038/s41598-023-46946-8

**Published:** 2023-11-09

**Authors:** Isaac Jardin, Sandra Alvarado, Vanesa Jimenez-Velarde, Joel Nieto-Felipe, Jose J. Lopez, Gines M. Salido, Tarik Smani, Juan A. Rosado

**Affiliations:** 1 Department of Physiology (Cellular Physiology Research Group), Institute of Molecular Pathology Biomarkers (IMPB), Universidad de Extremadura, 10003 Caceres, Spain; 2 Instituto de Biomedicina de Sevilla (IBiS), Hospital Universitario Virgen del Rocío/CSIC/Universidad de Sevilla, Seville, Spain; 3 Departamento de Fisiologia Medica y Biofisica, Facultad de Medicina, Universidad de Sevilla, Seville, Spain

**Keywords:** Breast cancer, Biochemistry, Cell biology

## Abstract

Orai1 is the pore-forming subunit of the Ca^2+^-release activated Ca^2+^ channels that mediate store-operated Ca^2+^ entry (SOCE) in excitable and non-excitable cells. Two Orai1 forms have been identified in mammalian cells, the full-length variant Orai1α, and the short form Orai1β, lacking the N-terminal 63 amino acids. Stem cells were isolated from non-tumoral breast epithelial cells of the MCF10A cell line, and the most representative ER+ , HER2 or triple negative breast cancer cell lines MCF7, SKBR3 and MDA-MB-231, respectively. Orai and TRPC family members expression was detected by RT-PCR and Western blotting. Changes in cytosolic Ca^2+^ concentration were analyzed by confocal microscopy using Fluo 4 and the spheroid-forming ability and self-renewal was estimated in culture plates coated with pHEMA using a cell imaging system. Here, we have characterized the expression of Orai family members and several TRPC channels at the transcript level in breast stem cells (BSC) derived from the non-tumoral breast epithelial cell line MCF10A and breast cancer stem cells (BCSC) derived from the well-known estrogen receptor positive (ER+), HER2 and triple negative cell lines MCF7, SKBR3 and MDA-MB-231, respectively. Furthermore, we have evaluated the mammosphere formation efficiency and self-renewal of the BSC and BCSC. Next, through a combination of Orai1 knockdown by iRNA and the use of MDA-MB-231 KO cells, missing the native Orai1, transfected with plasmids encoding for either Orai1α or Orai1β, we show that Orai1 is essential for mammosphere formation and self-renewal efficiency in BCSC derived from triple negative and HER2 subtypes cell cultures, while this channel has a negligible effect in BCSC derived from ER+ cells as well as in non-tumoral BSC. Both, Orai1α, and Orai1β support SOCE in MDA-MB-231-derived BCSC with similar efficiency, as well as COX activation and mammosphere formation. These findings provide evidence of the functional role of Orai1α and Orai1β in spheroid forming efficiency and self-renewal in breast cancer stem cells.

## Introduction

Store-operated Ca^2+^ entry (SOCE) is an ubiquitous mechanism for Ca^2+^ influx that participates in a large number of physiological processes from cellular functions such as lactation^1^, myoblast differentiation and excitation-contraction coupling^2^, platelet aggregation^3^ and B cells metabolic reprogramming^4^ to subcellular events such as the maintenance of regenerative Ca^2+^ oscillations^5^ or the regulation of gene transcription^6^. Occupation of G-protein coupled receptors by physiological agonists leads to the generation of IP_3_, which, in turn, activates the release of stored Ca^2+^ from the endoplasmic reticulum (ER) and the subsequent store-operated entry of Ca^2+^ predominantly through CRAC (Ca^2+^-release activated Ca^2+^) channels^7^. A decrease in ER Ca^2+^ concentration is detected by the ER Ca^2+^ sensors STIM1 (stromal interaction molecule 1) and/or its lower Ca^2+^ affinity homolog, STIM2, leading to both, clustering of STIM proteins at ER/plasma membrane (PM) junctions and a conformational change that results in the extension of the STIM C-terminal region facilitating the molecular interaction with and activation of the CRAC channels in the PM^8^. Native CRAC channels, which mediate the highly Ca^2+^ selective current *I*_CRAC_, consists of heterohexamers of all three Orai isoforms (Orai1-3)^9^ where Orai1 plays a predominant role while Orai2 and Orai3 modulate the extent of Ca^2+^ influx to match the Ca^2+^ signals to the magnitude of agonist stimulation^5^. Ca^2+^ influx through CRAC channels has been reported to induce PM translocation and activation of TRPC1 channels, leading to a store-dependent and less Ca^2+^ selective ion current called *I*_SOC_^10^.

In mammalian cells, Orai1 is present in two forms, the full-length variant Orai1α, containing 301 amino acids, and the short form Orai1β, generated from alternative translation initiation at methionine 64 in the long, Orai1α, form^11^. While both Orai1 variants supports the *I*_CRAC_ current^12^, they are non-redundant as several differences have been reported between them. Among them, Orai1α, exclusively, supports the Ca^2+^-selective current mediated by arachidonate and leukotriene C4, *I*_ARC_^12^, as well as NF-κB activation^6^ and interacts with the low Ca^2+^-affinity adenylyl cyclase 8^13–15^. Furthermore, Orai1α exhibits a greater sensitivity than Orai1β to fast Ca^2+^-dependent inactivation^12^ and, while Orai1α is essential for TRPC1 plasma membrane location and activation in all the cell types investigated^12^, the functional role of Orai1β in the activation of TRPC1/SOC channels is cell specific^16^.

Breast cancer cells have been reported to remodel the expression and function of a variety of Ca^2+^-handling proteins^17,18^, including Orai and TRPC channels^19,20^, which play relevant functional roles in the development of tumoral hallmarks such as enhanced proliferation, migration and apoptosis resistance. However, little is known about the phenotype remodelling exhibited by breast cancer stem cells, a small subpopulation of cells present in the breast tumours with low proliferative profile, resistance to chemotherapy and radiation and self-renewal ability, differentiation into heterogeneous cell lineages and high metastatic capability^21,22^. Here we report, for the first time, the expression profile of the Orai family members, Orai1-3, and different TRPC channels, TRPC1, 3 and 6, in breast cancer stem cells (BCSC) derived from the widely used estrogen receptor-positive (ER+) cell line MCF7, the HER2 cell line SKBR3 and the triple negative breast cancer (TNBC) cell line MDA-MB-231, as well as in breast stem cells (BSC) derived from the non-tumoral breast epithelial cell line MCF10A. Our results indicate that Orai1 plays an essential role in the mammosphere formation and self-renewal ability, distinctive features of stem cells, as well as COX activity of the subpopulation of BCSC derived from the different breast cancer subtypes, more precisely in those derived from TNBC. Furthermore, we show that Orai1α and Orai1β support TNBC-derived BCSC proliferation and spheroid formation with similar efficiency.

## Material and methods

### Ethical approval

Experimental approaches were approved by the local ethical committee (University of Extremadura and Extremadura Health Service, Spain). No animals were employed during the study.

### Reagents

Fluo-4 acetoxymethyl ester (AM) (F14201) was from Molecular Probes. High-glucose Dulbecco’s modified Eagle’s medium, fetal bovine serum, trypsin, penicillin/streptomycin, TRIzol reagent, qRT–PCR primers, high-capacity complementary DNA reverse transcription kit, SYBR Green PowerUp, Clean-Blot IP detection reagent, SuperSignal West Dura extended duration substrate reagent and Pierce BCA protein assay kit were purchased from Thermo Fisher Scientific (Madrid, Spain). Phenol red-free DMEM/F12 and B27 supplement were from Lonza (Porriño, Spain). Complete EDTA-free protease inhibitor cocktail tablets were from Roche Diagnostics GmbH. DharmaFECT kb transfection reagent was obtained from Cultek (Madrid, Spain). Carbachol, protein A agarose beads, HEPES (H3375), EGTA, EDTA, bovine serum albumin (BSA), sodium azide, dimethyl-BAPTA, arachidonic acid, rabbit polyclonal anti-Orai1 antibody (catalog number: O8264, epitope: amino acids 288–301 of human Orai1), and rabbit polyclonal anti-β-actin antibody (catalog number: A2066, epitope: amino acids 365–375 of human β-actin) were obtained from Sigma-Aldrich (Madrid, Spain). Cyclooxygenase (COX) Activity Assay Kit (ab204699) was purchased from Abcam (Madrid, Spain). Horseradish peroxidase–conjugated goat anti-mouse immunoglobulin G (IgG) antibody and goat anti-rabbit IgG antibody were from Jackson Laboratories (Bar Harbor, Maine, USA). TK-promoter and CMV-promoter Orai1α-enhanced GFP (eGFP) and Orai1β-eGFP plasmids were kindly provided by Professor Mohamed Trebak (Department of Pharmacology and Chemical Biology, University of Pittsburgh). All other reagents were of an analytical grade.

### Cell culture and transfections

MCF10A, MCF7, SKBR3 and MDA-MB-231 cell lines were obtained from the American Type Culture Collection (ATCC; Manassas, VA, USA), and cultured at 37 °C with a 5% CO2 in Dulbecco’s Modified Eagle Medium (DMEM) supplemented with 10% (v/v) fetal bovine serum and 100 U/mL penicillin and streptomycin (MCF7, SKBR3 and MDA-MB-231) or DMEM-F12, supplemented with 5% (v/v) horse serum, 10 μg/mL insulin, 0.5 μg/mL hydrocortisone, 100 ng/mL cholera toxin and 20 ng/mL epidermal growth factor (MCF10A). MDA-MB-231 cells with CRISPR-mediated knockout of Orai1 (Orai1-KO) were kindly donated by Mohamed Trebak. Cells were transfected with 1 µg/mL shOrai1, Orai1-GFP, Orai1α-GFP, Orai1β-GFP or scramble plasmids using Dharmafect transfection reagent and were used 48 h after transfection. For shOrai1, the sense sequence was 5′-CACCTCACTGGTTAGCCATAAGACGAATCTTATGGCTAACCAGTGA-3′, and the antisense sequence was 5′-AAAACCTTTACACGCTAGATGGTTTGCTCTTATGGCTAACCAGTGA- 3′.

### Quantitative RT-PCR

Total RNA isolation and single-strand cDNA synthesis was performed in spheroids derived from breast cancer stem-like cells (BSC). The primers used are depicted in Table 1. SYBR green qRT-PCR was performed using SYBR® Premix Ex Taq™ (Takara Bio Inc., Otsu, Shiga, Japan) in an Applied Biosystems STEPONE Real-Time thermal cycler (Life Technologies Corporation, Carlsbad, CA) as described previously. PCR products were obtained using the following cycling conditions: 96 °C for 2 min, followed by 35 cycles of 96 °C for 15 s, 48–56 °C for 25 s and finished with 72 °C for 10 min. mRNA abundance was calculated by the comparative CT (ΔΔCT) method using the equation: RQ=2−ΔΔCT. The amount of mRNA transcripts was normalized to GAPDH expression and represented as mean expression relative to MCF10A BSC specific mRNA ± S.E.M.

**Table 1:** Primers used in qRT-PCR.

Protein	Forward primer	Reverse primer
hOrai 1	AGCAACGTGCACAATCTCAA	GTCTTATGGCTAACCAGTGA
hOrai 2	CGGCCATAAGGGCATGGATT	TTGTGGATGTTGCTCACGGC
hOrai 3	CTCTTCCTTGCTGAAGTTGT	CGATTCAGTTCCTCTAGTTC
hTRPC1	TGCGTAGATGTGCTTGGGAG	ATGCTCTCAGAATTGGATCC
hTRPC3	GGAAGGACTGTAAAGGACA	CACAACGGAAGTCACTTCA
hTRPC6	TCATCATGGTGTTTGTGGC	GCAAAACAATGACCATTGTAA
GAPDH	GTCTCCTCTGACTTCAACAGCG	ACCACCCTGTTGCTGTAGCCAA

### Generation of spheroids derived from breast cancer stem-like cells

Mammospheres from cell cultures of the different subtypes of breast cells were generated using phenol red-free DMEM/F12 containing B27 supplement and SingleQuot^TM^ (Cambrex Bio Science, UK). Adherent cells were seeded in non-adherent plates (culture plates coated with pHEMA). After 5–7 days the number of mammospheres between 50 and 100 µm diameter were counted and the mammosphere forming efficiency and self-renewal under the different conditions was calculated as previously described: number of mammospheres per well/number of cells seeded per well × 100^23^. Bright-field images were taken on a EVOS FL Auto 2 Cell Imaging System (Thermofisher, Spain) using a long working distance Plan- Apochromat 10 × 0.25 at a zoom of 1.3×. Mammosphere forming efficiency and self-renewal were calculated by using Fiji ImageJ software v.1.8.0_172 (NIH, Bethesda, MD, USA). By using CD44^+^/CD24^-^ cellular subset via FACS using anti-CD24-phycoerythrin (PE) and anti-CD44-fluorescein isothiocyanate (FITC) monoclonal antibodies, we confirmed the stem-like phenotypes of the isolated cells (MCF10A: 81.62 ± 0.36%; MCF7: 85.45 ± 0.50%; SKBR3: 93.74 ± 1.09% and MDA-MB-231: 95.68 ± 0.54%)^24^. In cells treated with synta66, 100 µM synta66 was added to the medium during the mammosphere formation period.

### Western blotting

Western blotting was performed as described previously^6^. Briefly, cells cultured on 100-mm Petri dish (8 × 10^6^ cells) were detached and resuspended in HEPES-buffered saline (HBS) pH 7.4, containing 125 mM NaCl, 5 mM KCl, 1 mM MgCl_2_, 5 mM glucose, and 25 mM HEPES, supplemented with 0.1% (w/v) BSA. Cells were kept at 37 °C for 15 min. Next, cells were stimulated with 2 μM TG or with vehicle and subsequently lysed with ice-cold 2×NP-40 buffer, pH 8, containing 137 mM of NaCl, 20 mM of Tris, 2 mM of EDTA, 10% glycerol, 1% Nonidet P-40, 1 mM of Na_3_VO_4_, and complete EDTA-free protease inhibitor tablets. After 20-30 min at 4 °C, cell lysates were centrifugated at 16,000×*g* for 15 min and the supernatant was transferred to a fresh tube. Afterwards, the protein concentration was determined by using the BCA Protein Assay kit and, subsequently, equal volume 2×Laemmli´s sample buffer was added. Cell lysates were resolved by 10% or 12% SDS-PAGE and separated proteins were electrophoretically transferred onto nitrocellulose membranes for subsequent probing. Blots were incubated overnight with 10% (w/v) BSA in Tris-buffered saline with 0.1% Tween-20 (TBST) to block residual protein binding sites. Immunodetection of Orai1 and β-actin was achieved by incubation for 1 h with anti-Orai1 antibody diluted 1:1000 in TBST and overnight with anti-β-actin antibody diluted 1:2000 in TBST^6^. The primary antibody was removed, and blots were washed six times for 5 min each with TBST. To detect the primary antibody, blots were incubated for 1 h with horseradish peroxidase-conjugated goat anti-mouse IgG antibody, horseradish peroxidase-conjugated goat anti-rabbit IgG antibody diluted 1:10000 in TBST and then, exposed to enhanced chemiluminiscence reagents for 5 min. The antibody binding was assessed with a ChemiDoc Imaging System (Bio-Rad, Madrid, Spain) and the density of bands was measured using Fiji ImageJ software v.1.8.0_172 (NIH, Bethesda, MD, USA). Data were normalized to the amount β-actin from the same gel.

### Determination of cytosolic free-Ca^2+^ concentration ([Ca^2+^]i)

Spheroids derived from breast cancer stem-like cells were loaded with fluo-4 by incubation with 2 μM fluo-4/AM for 45 min at 37 °C. Poly-L-Lysine coated coverslips with cultured spheroids were mounted on a perfusion chamber and placed on the stage of a LSM900 confocal microscope (Carl Zeiss, Germany) using a LD LCI Plan-Apochromat 25×/0.8 multi-immersion objective, with water, at a zoom of 1.3×. Fluo-4 was excited with the 488 nm line of 10 mW diode laser at 1–4% power to minimalize the bleaching of the sample during monitoring. Fluorescence emission was detected between 492 and 602 nm by an Airyscan detector. The pinhole was set at 1 Airy Unit (23.5099 pixels per micron resolution) and no line averaging was used. The detection gain 700V was chosen such that the fluo-4 fluorescence was at 15-20% of the dynamic level of the detectors. Cells were continuously superfused at room temperature with HBS, pH 7.4, supplemented with 0.1% (w/v) BSA and 1.8 mg/mL glucose. Fluo-4 fluorescence was recorded with ZenBlue 3.4 acquisition and analysis software from Zeiss. Fluorescence mobilization was calculated pixel by pixel with Fiji ImageJ software v.1.8.0_172 (NIH, Bethesda, MD, USA), and the data were presented as arbitrary units (a.u.). Histamine-evoked changes in [Ca^2+^]_i_ were estimated as the area under the curve measured as the integral of the rise in fluo-4 fluorescence 2.5 min after the addition of the agonist and taking a sample every 5 s.

### Cyclooxygenase (COX) activity assay

COX activity was assessed as described by the manufacturer (ab204699, Abcam). Briefly, 2 × 10^6^ cells breast cancer stem-like cells were harvested, centrifuged at 200×*g* for 5 min, resuspended in cold PBS, and subsequently lysed with ice-cold 2×NP-40 buffer, pH 8. After 20–30 min at 4 °C, cell lysates were centrifugated at 16,000×*g* for 15 min and the supernatant was transferred to a fresh tube. 20 µL of sample, 68 µL reaction mix and either 2 µL of COX specific inhibitor (SC560/COX-1 or Celecoxib/COX-2) or DMSO as vehicle were mixed in a 96-well plate. Each sample was mixed in three parallel wells. Next, Arachidonic Acid was added into each well to initiate the reaction, according to the manufacturer's instructions. Subsequently, fluorescence was recorded (Ex/Em = 535/587 nm) immediately in a kinetic mode once every 15 s for 30 min using a Varioskan Lux (Thermo Fisher Scientific).

### Statistical analysis

All data are presented as the mean ± standard error of mean (SEM). Analysis of statistical significance was performed using GraphPad Prism v.8.4.3 (GraphPad Software, San Diego, CA, USA). Kruskal-Wallis test combined with Dunn´s post hoc test were used to compare the different experimental groups. For comparison between two groups Mann-Whitney U-test was used. All data with *p* < 0.05 was deemed significant; “ns” = nonsignificant.

## Results

### Expression of Orai and TRPC channels in breast cancer stem cells

Stem cells were isolated from non-tumoral breast epithelial cells of the MCF10A cell line, and the most representative and widely studied ER+, HER2 or triple negative breast cancer cell lines MCF7, SKBR3 and MDA-MB-231, respectively, as described in Methods. The stem phenotype was confirmed by the expression of CD44 and CD24 as described in Methods, and the expression of Orai1, Orai2 and Orai3 in the isolated stem cells was analyzed at the transcript level by qRT-PCR. As shown in Fig. 1A, we have found that the breast cancer stem cells (BCSC) derived from MCF7, SKBR3 and MDA-MB-231 breast cancer cell lines overexpressed Orai1 and Orai2 as compared to breast stem cells (BSC) derived from the non-tumoral cell line MCF10A (*p* < 0.001; *n*=6). Intriguingly, analysis of Orai1 protein expression by Western blotting revealed that Orai1 is overexpressed in MCF7 and MDA-MB-231-derived BCSC but not in BCSC isolated from the SKBR3 cell line (Fig. 1B; *p* < 0.0001; *n*=6). Further analysis of the Orai1 expression in non-stem MCF10A, MCF7, SKBR3 and MDA-MB-231 cells in comparison with their corresponding BSC and BCSC revealed that Orai1 expression was significantly smaller in SKBR3 BCSC as compared to non-stem SKBR3 cells (*p* < 0.01; *n*=6) while its expression was similar between stem and non-stem cells in the remaining cell models (Fig. 1B). As depicted in Fig. 1A, Orai3 expression was enhanced at the transcript level in BCSC derived from MCF7 and MDA-MB-231 cell lines but attenuated in SKBR3-derived BCSC as compared to MCF10A-derived BSC (Fig. 1A; *p* < 0.001; *n*=6).

**Figue 1:**
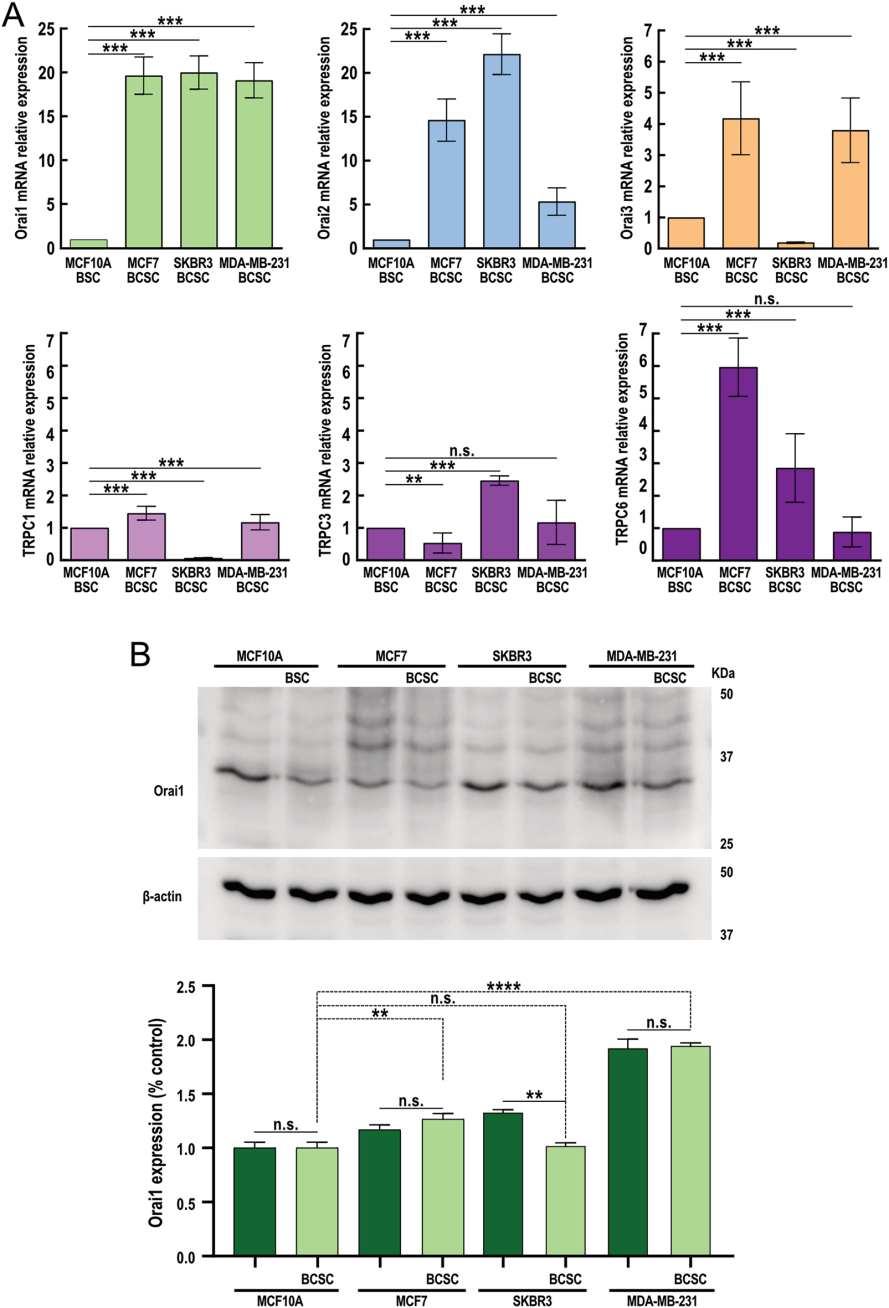
Expression of Orai and TRPC members in breast stem cells and breast cancer stem cells derived from the MCF10A, MCF7, SKBR3 and MDA-MB-231 cell lines. (**A**) RT-qPCR expression analysis of Orai1, Orai2, Orai3, TRPC1, TRPC3 and TRPC6 mRNA transcripts in BSC derived from the MCF10A cell line and BCSC derived from the MCF7, SKBR3 and MDA-MB-231 cell lines. Values were normalized to GAPDH expression and represented as mean expression relative to MCF10A-derived BCSC ± S.E.M.; n = 6. (**B**) Whole cell lysates from non-stem cells and stem cells derived from MCF10A, MCF7, SKBR3 and MDA-MB-231 cell lines were subjected to 10% SDS-PAGE and Western blotting with specific anti-Orai1 antibody, as indicated. Blots were reprobed with anti-β-actin antibody for protein loading control. Bar graph represents Orai1 expression presented as mean ± SEM. Data were statistically analyzed using Kruskal–Wallis test with multiple comparisons (Dunn´s test). **p < 0.01 and ****p < 0.0001.

Furthermore, our results indicate that Orai2 mRNA relative expression is greater in SKBR3 than in other BCSC and significantly much higher than in MCF10A BSC (Fig. 1A; *p* < 0.001; *n*=6). We have further analyzed the Orai2 expression at the protein level in the BSC and BCSC investigated and, as shown in suppl. Fig. 1, we have found that the Orai2 expression at the protein level in SKBR3 BCSC is not significantly different from that in MCF10A BSC or the BCSC derived from MCF7 and MDA-MB-231 cells. We have only found significant differences between the Orai2 protein expression in MDA-MB-231 BCSC and MCF10A BSC (Supp. Fig. 1; *p* < 0.001; *n*=6). Interestingly, we have not found differences in the protein expression of Orai2 in stem cells as compared to their corresponding non-stem cells ((Supp. Fig. 1; *n*=6).

TRPC channels, such as TRPC1, TRPC3 and TRPC6 also play a relevant role in agonist induced Ca^2+^ entry. Hence, we have analyzed the expression of these channels at the transcript level in BCSC derived from the breast cancer cell lines MCF7, SKBR3 and MDA-MB-231 as compared to BSC derived from the non-tumoral cell line MCF10A. MCF7-derived BCSC exhibited a significant overexpression of TRPC1 and, remarkably, TRPC6, while the expression of TRPC3 was attenuated as compared to BSC derived from the MCF10A cell line (Fig. 1A; *p* < 0.01; *n*=6). BCSC isolated from the SKBR3 cell line show null expression of TRPC1 while overexpression TRPC3 and TRPC6 (Fig. 1A; *p* < 0.001; *n*=6); meanwhile, BCSC-derived from the triple negative breast cancer cell line MDA-MB-231 exhibit a significantly greater expression of TRPC1 (Fig. 1A; *p* < 0.001; *n*=6) while the expression of TRPC3 and TRPC6 was similar to that observed in MCF10A-derived BSC.

### Mammosphere formation efficiency and self-renewal of MCF10A-derived BSC and BCSC derived from MCF7, SKBR3 and MDA-MB-231 cell lines.

**Figure 2:**
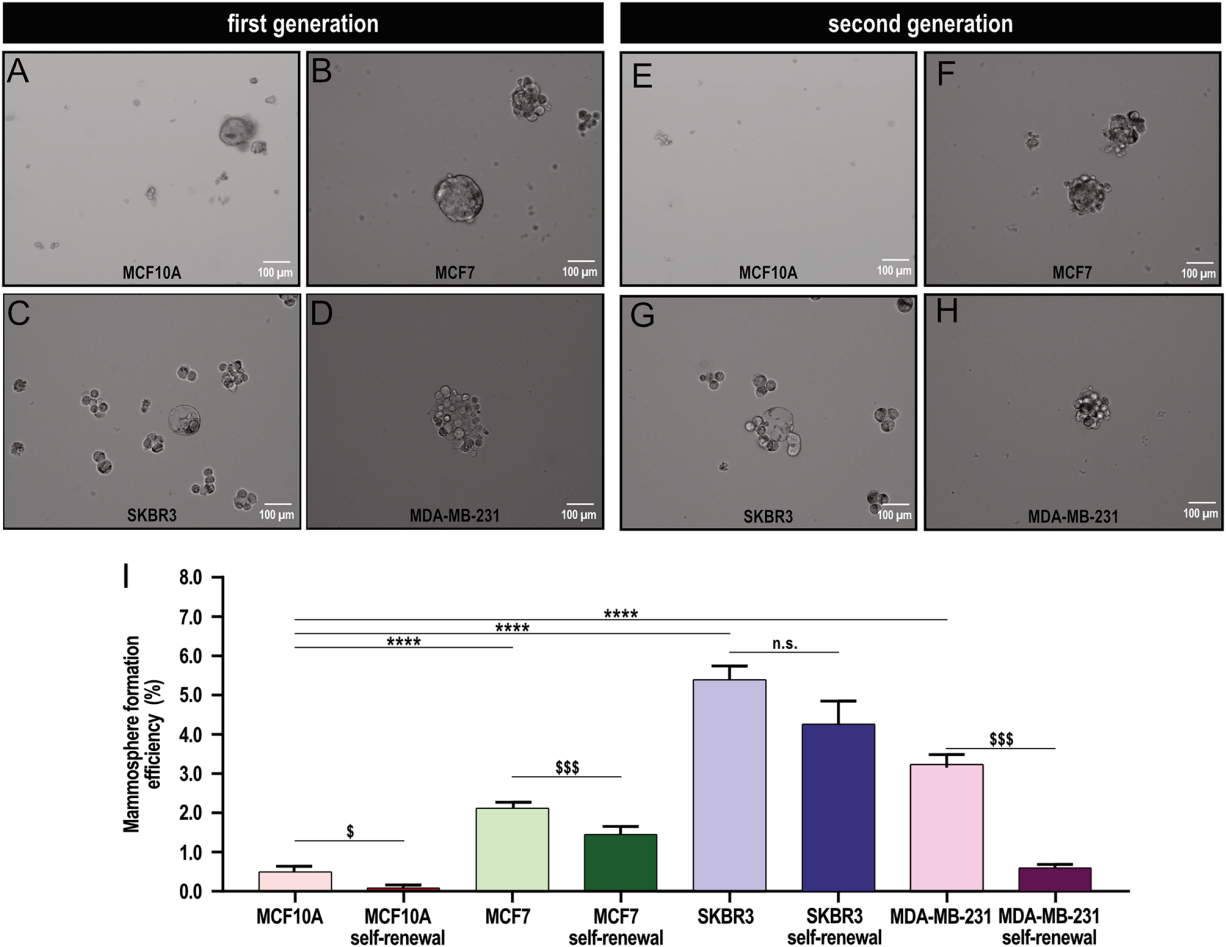
Sphere formation and self-renew stem cell efficiency in BSC derived from MCF10A and BCSC derived from MCF7, SKBR3 and MDA-MB-231 cell lines. Mammosphere formation assay (**A**–**D**) and self-renewal capacity (**E–H**) of MCF10A-derived BSC and BCSC derived from MCF7, SKBR3 and MDA-MB-231 cell lines. BSC and BCSC isolation, mammosphere formation and self-renewal was performed as described in Methods. **I** Bar graph represents mammosphere formation efficiency of stem cells derived from the different breast cancer and non-tumoral cell lines presented as mean ± SEM and expressed in percentage of the total cultured cells. Data were statistically analyzed using Mann–Whitney U test. ^$^*p* < 0.05 and ^$$$^*p* < 0.001 as compared to the efficiency to form first generation mammospheres. ^*^p < 0.05, ^**^p < 0.01 and ^***^p < 0.001 as compared to the efficiency to form first generation mammospheres in MCF10A-derived BSC.

The mammospheres were generated from the non-tumoral breast epithelial MCF10A cell line and the ER+, HER2 and triple negative breast cancer cell lines MCF7, SKBR3 and MDA-MB-231, respectively, in phenol red-free DMEM/F12 containing B27 supplement and SingleQuot^TM^. As shown in Fig. 2A–D, the cells derived from the different non-tumoral and tumoral cell lines efficiently formed compact mammospheres. The mammosphere formation efficiency was distinct in the different cell lines with non-tumoral cells showing the lowest efficiency and SKBR3 and MDA-MB-231 cell lines the highest (Fig. 2I). We further analyzed the formation of second generation mammospheres as indicative of self-renewal. As depicted in Fig 2E–I, the ability of MCF10A-derived BSC to form second generation mammospheres was almost null, meanwhile, BCSC isolated from MCF7, SKBR3 and MDA-MB-231 cell lines exhibited a significant ability to self-renew despite all the cell lines investigated showed a lower efficiency of secondary than primary mammosphere formation (Fig. 2I).

### Functional role of Orai1 in the mammosphere formation efficiency of MCF10A-derived BSC and BCSC derived from MCF7, SKBR3 and MDA-MB-231 cell lines

**Figure 3:**
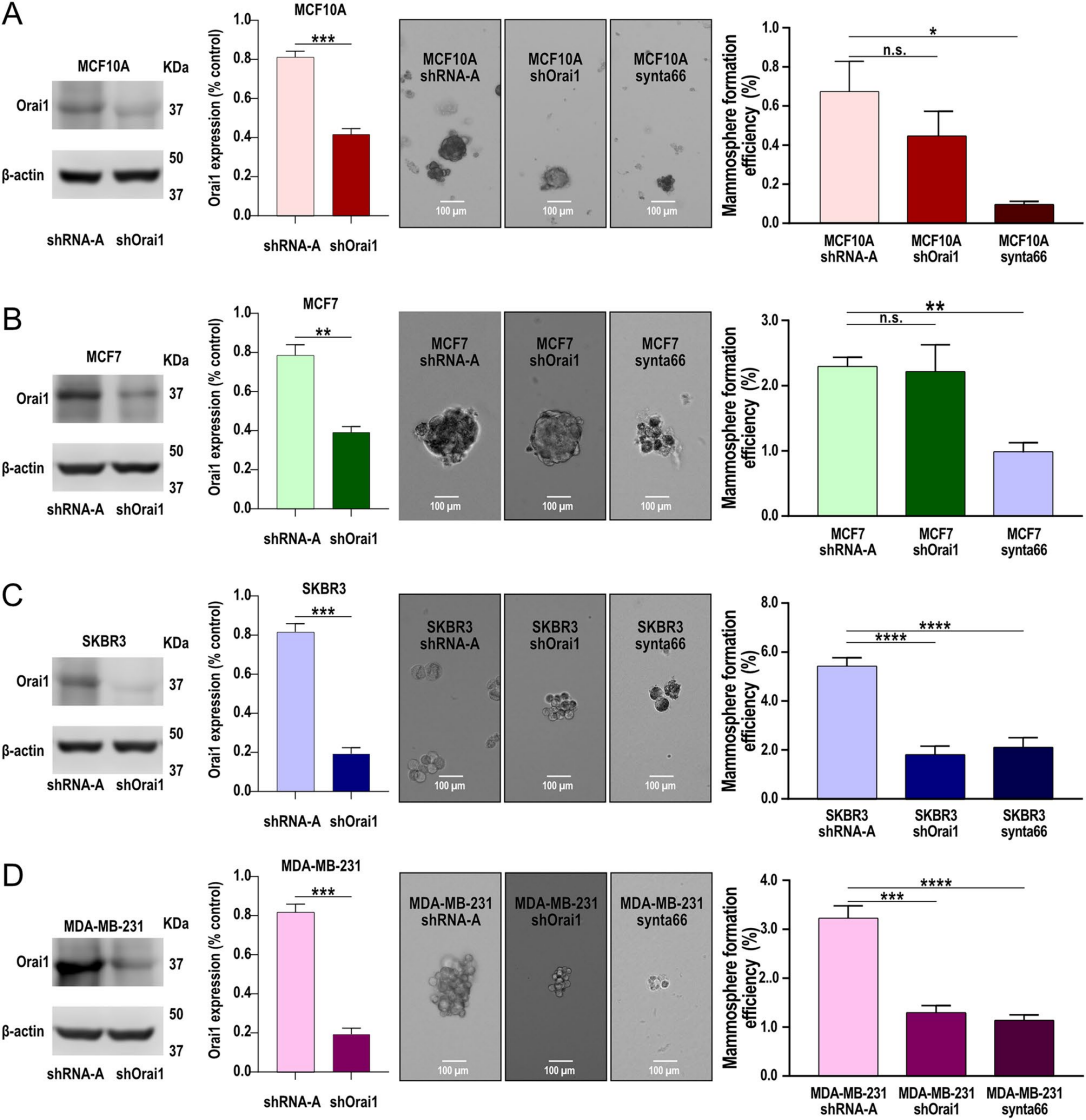
Functional role of Orai1 in mammosphere formation efficiency in BSC derived from MCF10A and BCSC derived from MCF7, SKBR3 and MDA-MB-231 cell lines. BSC were isolated from MCF10A (**A**) and BCSC derived from MCF7 (**B**), SKBR3 (**C**) and MDA-MB-231 (**D**) cell lines were isolated as described in Methods. Isolated BSC and BCSC were transfected with shOrai1 or empty vector (shRNA-A). Forty-eight hours later cells were lysed and then subjected to 10% SDS-PAGE and Western blotting with anti-Orai1 antibody. Membranes were reprobed with the anti-β-actin antibody for protein loading control. Molecular masses indicated on the right were determined using molecular-mass markers run on the same gel. Blots are representative of four separate experiments. Bar graph represents Orai1 expression under the different experimental conditions. Data were statistically analyzed using Mann–Whitney U test. **p < 0.01 and ***p < 0.001 as compared to Orai1 expression in cells transfected with shRNA-A. Isolated BSC and BCSC were transfected with shOrai1 and twenty-four hours after transfection cells were seeded in ultra-low attachment plates to perform the mammosphere formation assay. Alternatively, cells were culture in ultra-low attachment plates to analyze the mammosphere formation efficiency in the presence of 100 µM synta66 or the vehicle as control. Vehicle-treated cells (not shown) exhibited a similar mammosphere formation efficiency as cells transfected with shRNA-A. The mammosphere formation efficiency was estimated as described in Methods. Data were statistically analyzed using Mann–Whitney U test. *p < 0.05, **p < 0.01, ***p < 0.001 and ****p < 0.0001.

Orai1 is the key pore-forming subunit of the CRAC channels in MCF10A and MDA-MB-231 cells, where SOCE is strongly dependent on Orai1 expression and function^19,20^. By contrast, while Orai1 has been reported to be highly expressed in MCF7 cells, SOCE in this cell line strongly depends on Orai3^20,25^. As Orai1 is highly expressed, at the transcript and protein level, in breast cancer cells, with the exception of SKBR3, where we have found overexpression of the Orai1 mRNA but normal Orai1 protein expression (see Fig. 1), we have investigated the functional role of Orai1 in the mammosphere formation efficiency in BCSC derived from these cells as compared to BSC isolated from the MCF10A breast epithelial cell line. To assess the role of Orai1 in the mammosphere formation efficiency, MCF10A BSC and MCF7, SKBR3 and MDA-MB-231 BCSC were transfected with shRNA for Orai1 or scramble plasmids (shRNA-A). As shown in Fig. 3A–D, transfection with shOrai1 significantly attenuated the expression of the target protein by about 50–75% in 48 h (*p* < 0.01; Mann–Whitney U test).

As depicted in Fig. 3, cell transfection with shOrai1 significantly attenuated the efficiency of mammosphere formation in SKBR3 and MDA-MB-231 BCSC, while Orai1 knockdown slightly, but not significantly, decreased the ability of MCF10A BSC and MCF7 BCSC to form mammospheres. To further address the role of Orai1 in the mammosphere formation efficiency of the investigated cells we used the pharmacological Orai1 inhibitor synta66. As shown in Fig. 3, treatment of MCF10A BSC and MCF7, SKBR3 and MDA-MB-231 BCSC with 100 µM synta66 significantly attenuated their ability to form mammospheres, thus indicating that Orai1 plays a relevant role in the mammosphere formation efficiency in BSC and BCSC.

### Orai1 is essential for mammosphere formation and self-renewal in BCSC derived from the MDA-MB-231 cell line.

**Figure 4:**
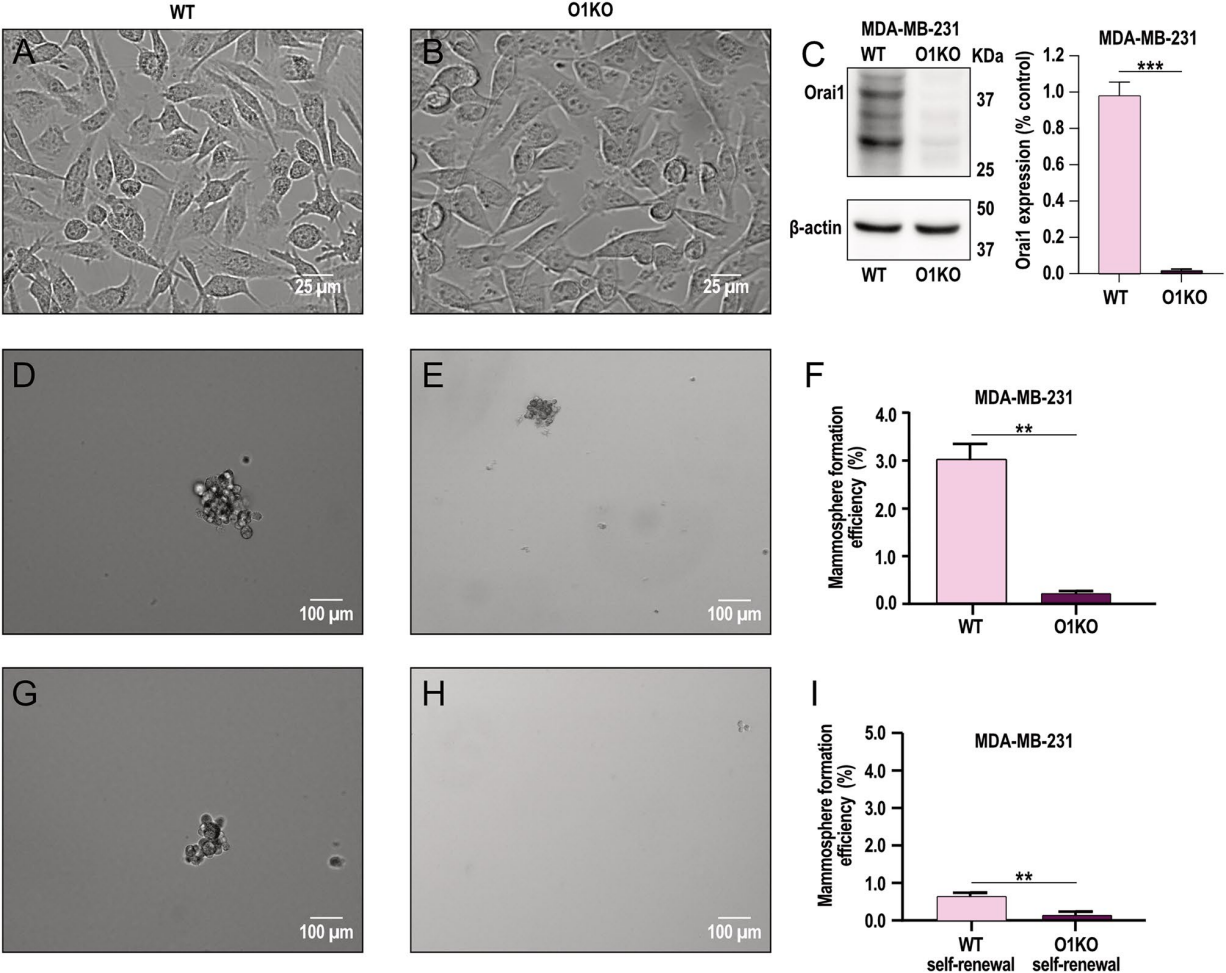
Sphere formation and self-renew stem cell efficiency in BCSC derived from Orai1-KO MDA-MB-231 cells. **A**-**C** BCSC derived from WT MDA-MB-231 cells (WT; **A**) and Orai1-KO MDA-MB-231 cells (O1KO; **B**) were lysed and then subjected to 10% SDS-PAGE and Western blotting with anti-Orai1 antibody. Membranes were reprobed with the anti-β-actin antibody for protein loading control. Molecular masses indicated on the right were determined using molecular-mass markers run on the same gel. Blots are representative of four separate experiments. Bar graph represents Orai1 expression in WT and O1KO MDA-MB-231 cells. Data were statistically analyzed using Mann–Whitney U test. ***p < 0.001 as compared to WT MDA-MB-231 cells. (**D**–**I**) Formation of first generation mammospheres (**D**–**F**) and self-renewal capacity (**G**–**I**) of BCSC derived from WT and O1KO MDA-MB-231 cells was performed as described in Methods. Bar graphs represent mammosphere formation efficiency presented as mean ± SEM and expressed in percentage of the total cultured cells. Data were statistically analyzed using Mann–Whitney U test. **p < 0.01 as compared to WT MDA-MB-231 cells.

We further analyzed the functional role of Orai1 in BCSC derived from Orai1 knockout MDA-MB-231 cells generated by CRISPR/Cas9 technology (O1KO). As depicted in Fig. 4A–C, O1KO MDA-MB-231 cells show a similar morphology than the parental MDA-MB-231 cells but did not express detectable amounts of Orai1 as determined by Western blotting (*p* < 0.001; *n* = 4). In O1KO BCSC the ability to form mammospheres was almost abolished as compared to WT BCSC, thus suggesting that Orai1 expression and function is required for MDA-MB-231 BCSC mammosphere formation (Fig. 4D–F, *p* < 0.01). Similarly, self-renewal efficiency was significantly attenuated in O1KO BCSC (Fig. 4G–I, *p *< 0.01). Altogether, these findings indicate that Orai1 plays an essential functional role in mammosphere formation and self-renewal in MDA-MB-231 BCSC.

**Figure 5:**
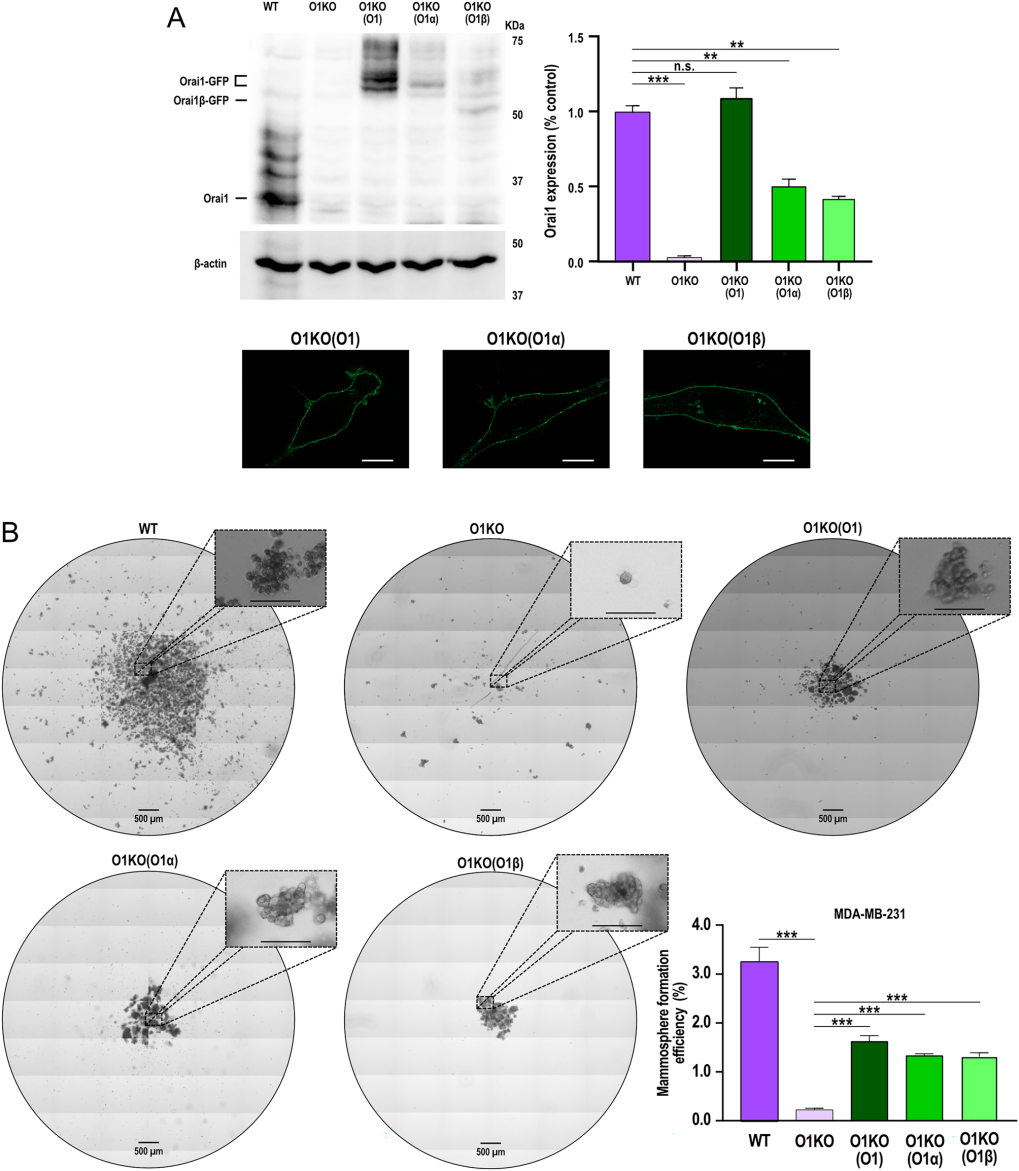
Functional role of Orai1α and Orai1β in the mammosphere formation efficiency of BCSC derived from Orai1 KO MDA-MB-231 cells. (**A**) (top) WT MDA-MB-231 cells, O1KO MDA-MB-231 cells and O1KO MDA-MB-231 cells transfected with either TK-promoter Orai1, Orai1α or Orai1β were lysed and subjected to Western blotting with anti-Orai1 antibody, followed by reprobing with anti-β-actin antibody for protein loading control. Molecular masses indicated on the right were determined using molecular-mass markers run on the same gel. Blots are representative of four separate experiments. Bar graph represents Orai1 expression under the different experimental conditions. Data were statistically analyzed using Kruskal–Wallis test with multiple comparisons (Dunn´s test). **p < 0.01 and ***p < 0.001 as compared to WT MDA-MB-231 cells. (**A**) (bottom) Representative confocal images of Orai1-GFP, Orai1α-GFP or Orai1β-GFP expressed in BCSC derived from Orai1-KO MDA-MB-231 cells. The scale bar represents 25 μm. (**B**) Formation of first generation mammospheres of BCSC derived from WT MDA-MB-231 cells, O1KO MDA-MB-231 cells and O1KO MDA-MB-231 cells transfected with either TK-promoter Orai1, Orai1α or Orai1β, as indicated, was performed as described in Methods. The scale bar in the box represents 100 μm. Bar graphs represent mammosphere formation efficiency presented as mean ± SEM and expressed in percentage of the total number of cells in the original WT MDA-MB-231 and transfected or untransfected O1KO MDA-MB-231 cell culture. Data were statistically analyzed using Kruskal–Wallis test with multiple comparisons (Dunn´s test). **p < 0.01 and ***p < 0.001 as compared to BCSC from O1KO MDA-MB-231 cells.

Two Orai1 variants have been identified in mammalian cells, the long form, Orai1α, the full-length Orai1, and the short form, Orai1β, generated by alternative translation initiation from methionine 64 in the Orai1α variant^11^. Hence, we have further explored the functional role of Orai1 variants in the mammosphere formation efficiency by expressing either Oraiα or Orai1β in BCSC derived from O1KO MDA-MB-231 cells. BCSC derived from O1KO MDA-MB-231 cells were transfected with either, Orai1 (expected to yield a mixture of both variants), Orai1α or Orai1β using expression plasmids carrying a thymidine kinase (TK) promoter. Transfected cells were analyzed for Orai1 expression by Western blotting and confocal microscopy. As shown in Fig. 5A (top panel) transfection with Orai1 fully rescued Orai1 expression, meanwhile, transfection with Orai1α or Orai1β partially rescued it. Cells transfected with Orai1, Orai1α or Orai1β exhibited fluorescence labelling confined exclusively at the plasma membrane (Fig. 5A, bottom panel). As mentioned above, BCSC derived from O1KO MDA-MB-231 cells exhibited a significantly smaller ability to form mammospheres that those derived from WT MDA-MB-231 cells (Fig. 5B; *p* < 0.001). Expression of either Orai1, Orai1α or Orai1β partially rescued the mammosphere formation efficiency in BCSC derived from O1KO MDA-MB-231 cells (*p* < 0.05; Fig. 1B; *p* < 0.01). As parental WT cells were treated the same as O1KO cells but using empty vector, and the Orai1 expression in WT and O1KO cells transfected with Orai1 was comparable we do not have an explanation for this difference. Interestingly, we did not detect significant differences in cells expressing either Orai1, Orai1α or Orai1β; therefore, these findings indicate that Orai1α and Orai1β equally support mammosphere formation in MDA-MB-231-derived BCSC.

### Orai1α and Orai1β are relevant for COX activation in BCSC derived from the MDA-MB-231 cell line

**Figure 6:**
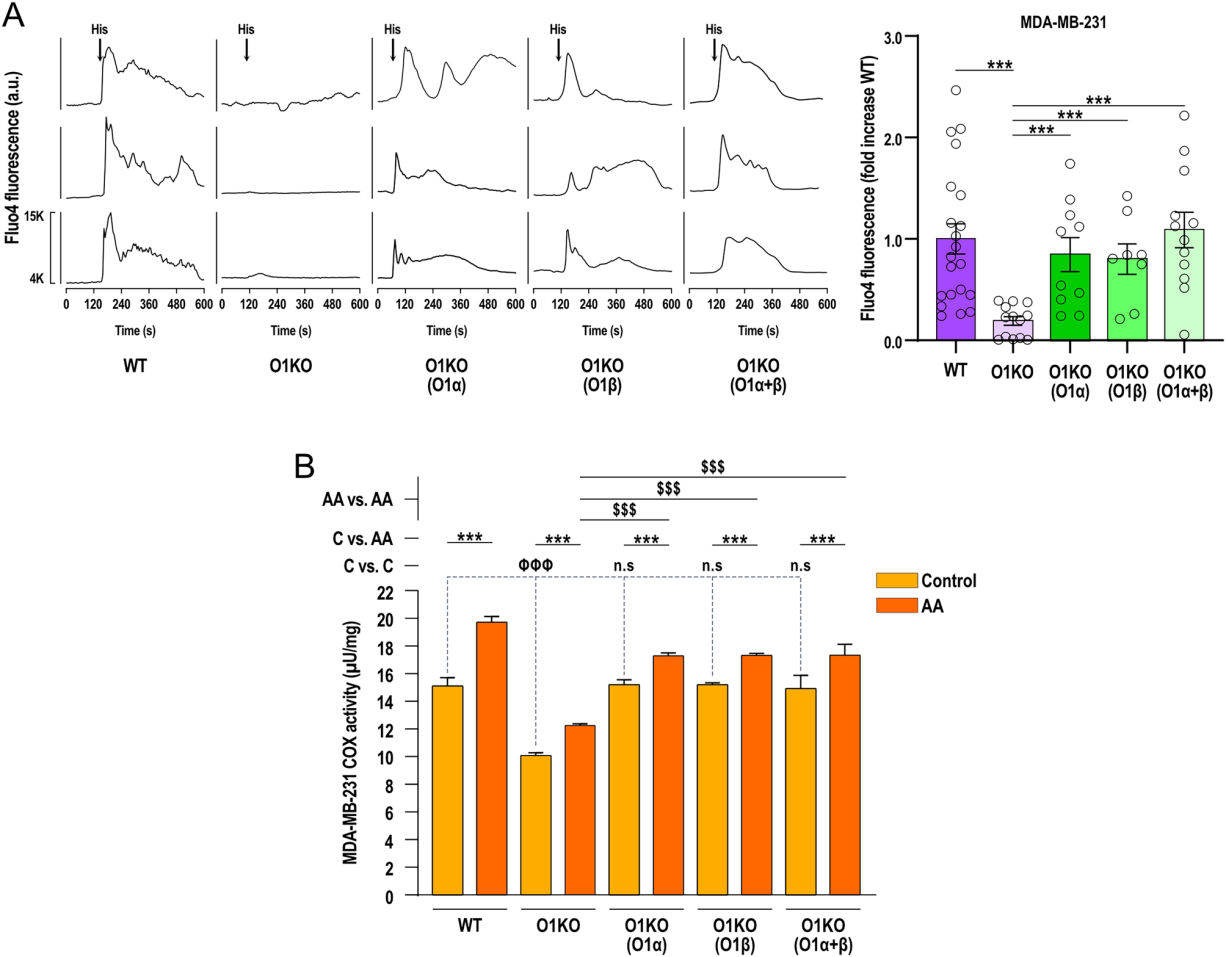
Functional role of Orai1α and Orai1β in agonist-induced Ca^2+^ mobilization and COX activation in BCSC derived from WT or Orai1 KO MDA-MB-231 cells. (**A**) BCSC were isolated from WT-MDA-MB-231 (WT) or Orai1-KO MDA-MB-231 cells either transfected with empty vector (O1KO) or with TK-promoter Orai1α (O1KO(O1α),TK-promoter Orai1β (O1KO(O1β) or both, as indicated. Representative Ca^2+^ mobilization in response to 100 μM histamine measured using fluo-4. Cells were superfused with HBS containing 1 mM Ca^2+^ and stimulated with 100 μM histamine (indicated by arrow). Representative traces from three cells/condition were chosen to represent the datasets (from left to right, n = 21, 13, 10, 8 and 12; n values correspond to individual mammospheres (8–10 cells analyzed per mammosphere)). Bar graphs are represented as mean ± SEM and were statistically analyzed using Kruskal–Wallis test with multiple comparisons (Dunn's test). ***p < 0.001 as compared to Ca^2+^ mobilization in BCSC derived from O1KO cells. (**B**) BCSC isolated from WT-MDA-MB-231 (WT) or Orai1-KO MDA-MB-231 cells either transfected with empty vector (O1KO) or TK-promoter Orai1α (O1KO(α)), Orai1β (O1KO(β)) or both (O1KO(α + β)), as indicated, were stimulated with arachidonic acid (AA; 8 μM) in a medium containing 1 mM Ca^2+^ and COX activity was determined as described in Methods. Bar graphs represent COX activity presented as mean ± SEM and expressed µU/mg. Data were statistically analyzed using Kruskal–Wallis test with multiple comparisons (Dunn´s test). ***p < 0.001 as compared to COX activity in non-stimulated cells. ^ΦΦΦ^*p* < 0.001 as compared to COX activity in O1KO cells.

We further explored the role of Orai1α and Orai1β on histamine-induced Ca^2+^ mobilization in BCSC derived from WT MDA-MB-231 cell cultures, O1KO MDA-MB-231 cell cultures, and O1KO MDA-MB-231 cells expressing either Orai1α or Orai1β. Traces from three representative cells are depicted in Fig. 6A. In the presence of 1 mM extracellular Ca^2+^, BCSC derived from WT MDA-MB-231 cells responded to 100 µM histamine with a transient increase in [Ca^2+^]_i_. By contrast, histamine-induced Ca^2+^ mobilization in O1KO BCSC was significantly attenuated (Fig. 6A traces and bar graph; *p* < 0.001). Transfection of O1KO BCSC with Orai1α, Orai1β or both expression plasmids rescued histamine-evoked Ca^2+^ mobilization (Fig. 6A traces and bar graph; *p* < 0.001 as compared to O1KO cells), which reveals the relevant role of both Orai1 variants in histamine-induced Ca^2+^ mobilization in BCSC derived from MDA-MB-231 cells.

COX has been reported to play a relevant role in cancer stem cell activity, promoting metastasis of cancer cells and apoptosis resistance^26^. Hence, we have evaluated the role of Orai1α and Orai1β on COX activity in BCSC derived from WT MDA-MB-231 cells, O1KO MDA-MB-231 cells, and O1KO MDA-MB-231 cells expressing either Orai1α or Orai1β. BCSC were stimulated with 8 μM arachidonic acid (AA) to induce COX activation. COX activity was determined using a COX activity assay as described in Methods. As shown in Fig. 6B, BCSC derived from WT MDA-MB-231 cells exhibit a detectable COX activity in resting conditions, which was significantly enhanced upon stimulation with AA (*p* < 0.001; *n* = 4). In O1KO BCSC both resting and stimulated COX activity were significantly attenuated (*p* < 0.001) despite AA was able to significantly enhance COX activity in these cells (Fig. 6B; *p* < 0.001; *n* = 4). Transfection of O1KO BCSC with Orai1α, Orai1β or both expression plasmids rescued COX activity in resting conditions as compared to O1KO BCSC (*p* < 0.001). Under these circumstances, AA was able to significantly enhance COX activity (Fig. 6B; *p* < 0.001; *n* = 4). These findings indicate that Orai1α and Orai1β play a relevant role in COX activation in BCSC derived from MDA-MB-231 cells.

## Discussion

Orai1 is a major pore-forming subunit of the CRAC channels that plays relevant functional roles, from lactation^1^ and immunity^27^ to apoptosis^28^ or myocardial electromechanical activity^29^. In breast ductal cells, Orai1 has been reported to play an essential role in Ca^2+^ influx^19,20^. Similarly, SOCE in triple negative breast cancer cells is strongly dependent on Orai1 and, subsequently, the development of a variety of cancer hallmarks, such as cell proliferation, migration or apoptosis resistance^19,20,30^. Here, we show that Orai1 plays an essential role for agonist-induced Ca^2+^ entry in triple negative breast cancer-derived BCSC, which is required for spheroid-forming efficiency and COX activity in these cells.

Isolated BCSC from the three major breast cancer molecular subtypes, i.e. ER+, HER2 and triple negative, exhibit an Orai and TRPC channel expression signature that, as compared to BSC derived from non-tumoral breast epithelial cells, qualitatively differs from their expression in differentiated cancer cells of the same subtype. For instance, at the transcript level Orai2 expression in ER+ MCF7 and triple negative MDA-MB-231 cell lines was comparable to that in non-tumoral MCF10A cells^31^ meanwhile in MCF7 and MDA-MB-231-derived BCSC Orai2 was overexpressed as compared to BSC-derived from the MCF10A cell line. Similarly, TRPC6 has been reported to be overexpressed in MDA-MB-231 cells^19,32^ while MDA-MB-231-derived BCSC shows a similar TRPC6 expression to BSC derived from the MCF10A cell line; by contrast, MCF7 and SKBR3-derived BCSC exhibit a marked TRPC6 overexpression.

Orai1 knockdown in BSC derived from MCF10A and the three BCSC using specific shRNA Orai1 has revealed that Orai1 is required for SKBR3 and MDA-MB-231-derived BCSC spheroid-forming efficiency. By contrast, Orai1 does not play a functional role in mammosphere formation in MCF10A and MCF7-derived stem cells. Nevertheless, cell treatment with the pharmacological Orai1 inhibitor synta66 significantly attenuated the ability of MCF10A BSC as well as MCF7, SKBR3 and MDA-MB-231 BCSC to form mammospheres. These findings indicate that Orai1 plays an important role in the mammosphere formation efficiency of these cells. A possible explanation to the different effects observed after treatment with synta66 or transfection with shRNA Orai1 in MCF10A BSC and MCF7-derived BCSC might reside in the smaller efficiency of Orai1 silencing in these cells. In addition, in MCF7-derived BCSC other Ca^2+^ permeable channels might also participate in the influx of Ca^2+^ required for spheroid formation. The latter is consistent with a predominant role of Orai3 in SOCE and function in differentiated ER + breast cancer cells^19,20^. Our findings are consistent with the relevant role of Orai1 in MCF10A and MDA-MB-231 cell migration^33^. The functional role of Orai1 in MDA-MB-231-derived BCSC was further confirmed in cells lacking Orai1 generated by CRISPR (O1KO BCSC), which provide a clean background, where the ability to form mammospheres and self-renew was abolished revealing the critical role for Orai1 in MDA-MB-231-derived BCSC function. In O1KO BCSC, expression of Orai1 (which is expected to yield both Orai1 forms), Orai1α or Orai1β using plasmids with the relatively week TK promoter in order to achieve protein expression close to the endogenous levels, completely rescued agonist-induced Ca^2+^ mobilization and COX activity, but, surprisingly, this procedure only partially overcame mammosphere forming efficiency. At present, we do not have an explanation for the different observations in O1KO BCSC expressing Orai1 as compared to BCSC derived from the parental MDA-MB-231 cell line. Our results indicate that Orai1, as well as its variants, is expressed at/by the plasma membrane and that SOCE is similar in WT BCSC and in Orai1α or Orai1β-expressing O1KO BCSC, thus suggesting that this procedure is not significantly altering the Orai1:STIM1 stoichiometry. We observed that BCSC derived from O1KO MDA-MB-231 cells exhibit a similar expression of Orai3, STIM1 and STIM2, but greater Orai2 expression than BCSC-derived from WT-MDA-MB-231 cells (Suppl. Fig. 2). At the moment we do not know the functional relevance of the Orai2 overexpression in these cells as SOCE (Fig. 6) and mammosphere formation (Figs. 4 and 5) are abrogated in BCSC derived from O1KO cells, which is consistent with the essential role of Orai1 as a component of the CRAC channels^9^.

## Conclusions

**Figure 7:**
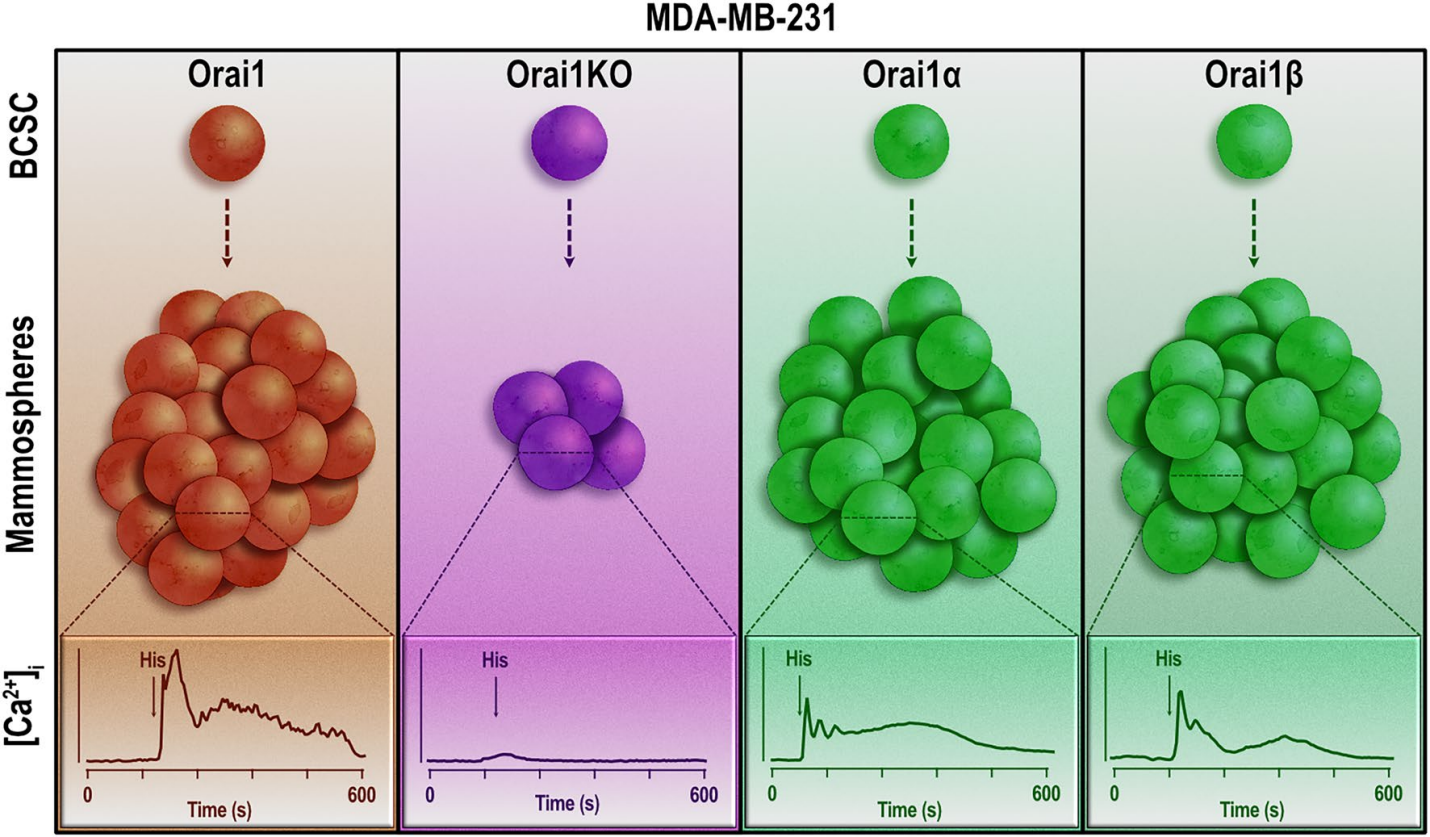
Overview of the role of Orai1 in BCSC spheroid formation. MDA-MB-231-derived breast cancer stem cells (BCSC) overexpress Orai1 and exhibit a significant agonist-evoked Ca^2+^ mobilization, leading to an efficient spheroid (mammosphere) formation. BCSC derived from Orai1-deficient cells shows attenuated agonist-induced Ca^2+^ mobilization, as well as spheroid-forming capability, thus revealing the functional role of Orai1 in these cells. Expression of Orai1α or Orai1β in Orai1-deficient BCSC rescue both histamine-stimulated Ca^2+^ mobilization and the spheroid-forming ability with similar efficiency, which indicates that both Orai1 variants support BCSC function equally well.

Our results reveal critical differences in the Orai and TRPC channel expression signature between BCSC and the differentiated (non-stem) breast cancer cells which offer the possibility of looking for new biomarkers that serve as a basis for the development of diagnostic and therapeutic strategies. In this context, Orai1 is overexpressed in BCSC derived from the most representative cell lines of the different breast cancer molecular subtypes and plays an essential role in SOCE, COX activation and spheroid-forming ability, with Orai1α and Orai1β supporting these cellular processes with the same efficiency (Fig. 7).

### Supplementary Information

Supplementary Figures.

## Data Availability

All the data used are provided in this article and are available upon reasonable written request. Please contact Isaac Jardin (ijp@unex.es) or Juan A. Rosado (jarosado@unex.es).
