# Supplementary material for: Orai1α and Orai1β support calcium entry and mammosphere formation in breast cancer stem cells
Source: Sci Rep. 2023 Nov 9;13:19471. doi: 10.1038/s41598-023-46946-8 (PMC10636192; doi:10.1038/s41598-023-46946-8)
Supplement: Supplementary file 1 — Supplementary Figures. [file 41598_2023_46946_MOESM1_ESM.docx]

Supplementary figures

Orai1α and Orai1β support calcium entry and mammosphere formation in breast cancer stem cells

## Isaac Jardin^1^, Sandra Alvarado^1^, Vanesa Jimenez-Velarde^1^, Joel Nieto-Felipe^1^, Jose J Lopez^1^, Gines M. Salido^1^, Tarik Smani^2,3^ and Juan A. Rosado^1^


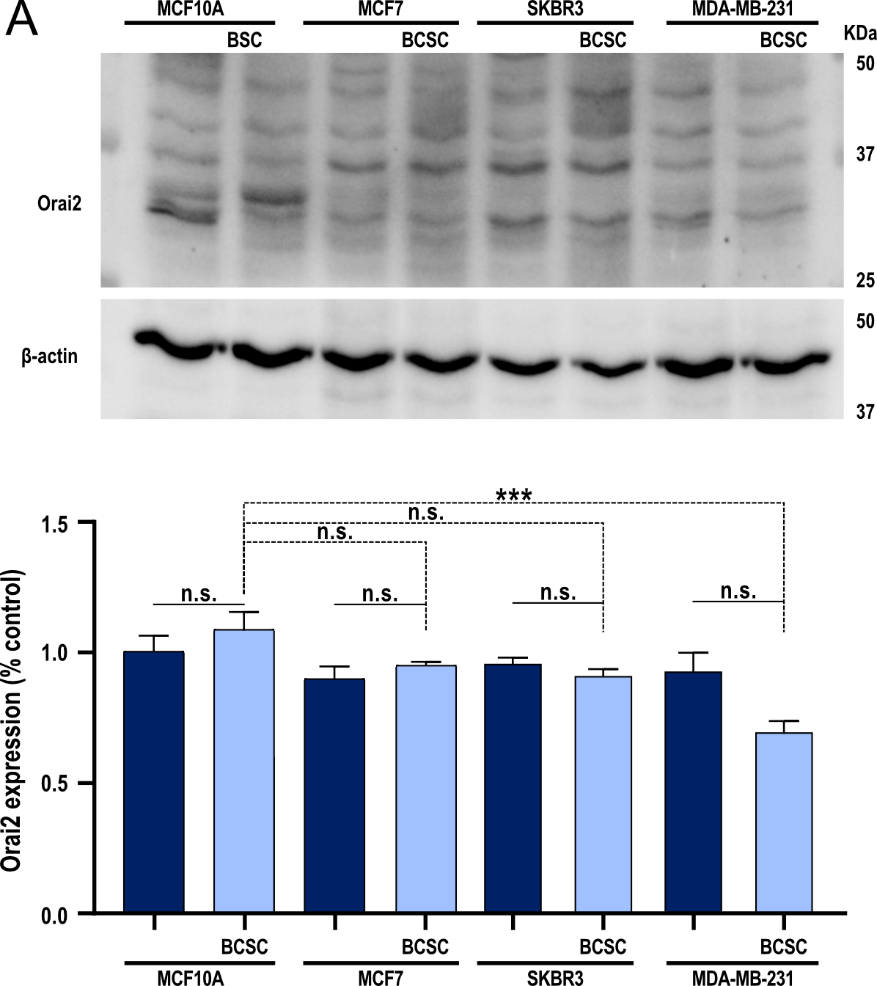


**Supplementary Fig. 1.** Expression of Orai2 protein in breast stem cells and breast cancer stem cells derived from the MCF10A, MCF7, SKBR3 and MDA-MB-231 cell lines. Whole cell lysates from non-stem cells and stem cells derived from MCF10A, MCF7, SKBR3 and MDA-MB-231 cell lines were subjected to 10% SDS-PAGE and Western blotting with specific anti-Orai2 antibody, as indicated. Blots were reprobed with anti-β-actin antibody for protein loading control. Bar graph represents Orai2 protein expression presented as mean ± SEM of 6 independent experiments. Data were statistically analyzed using Kruskal–Wallis test with multiple comparisons (Dunn´s test). ****p* < 0.001


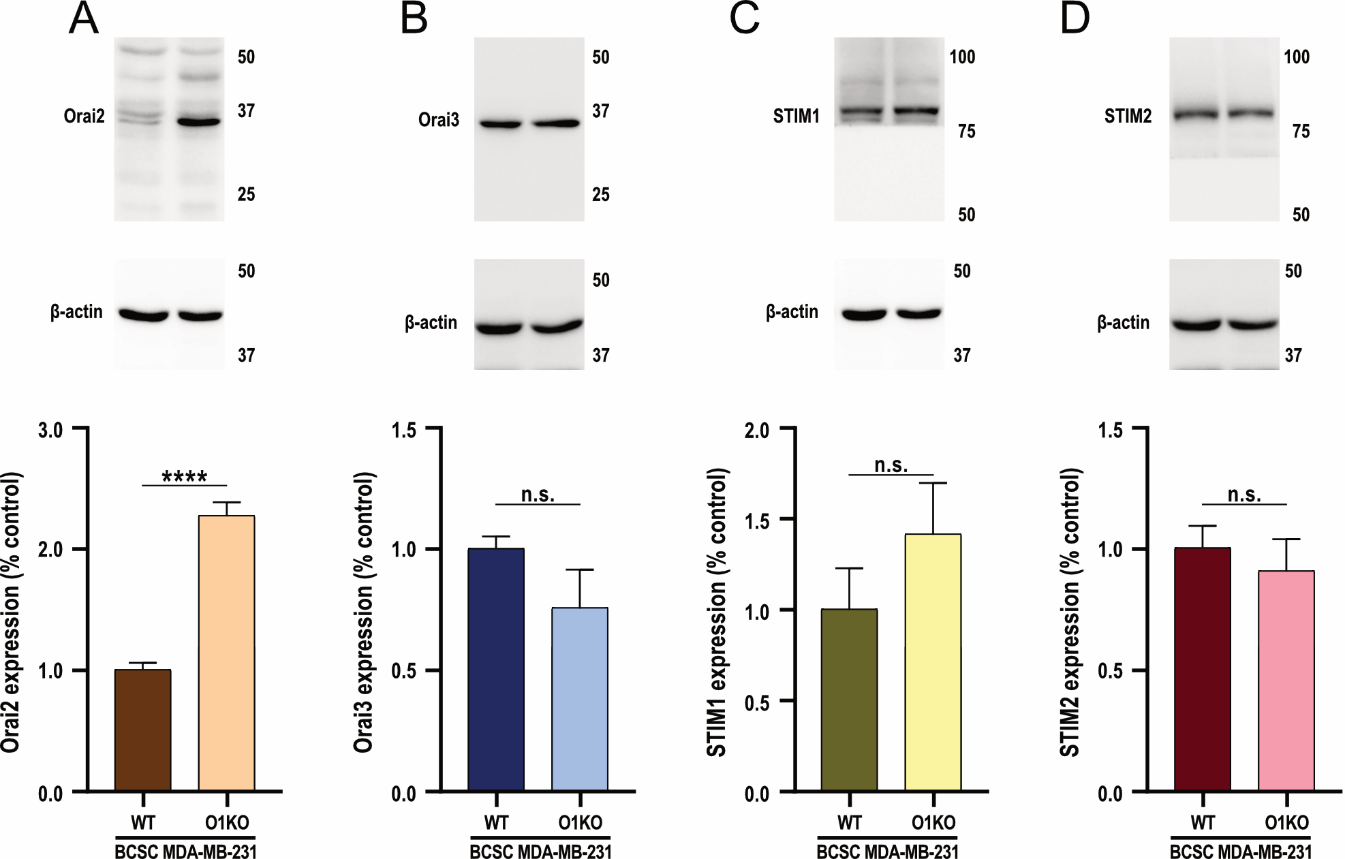


**Supplementary Fig. 2.** Expression of Orai2, Orai3, STIM1 and STIM2 in breast cancer stem cells (BCSC) derived from WT MDA-MB-231 and Orai1-KO MDA-MB-231. Whole cell lysates from stem cells derived from WT and Orai1-KO (O1KO) MDA-MB-231 cells were subjected to 10% SDS-PAGE and Western blotting with specific anti-Orai2, anti-Orai3, anti-STIM1 and anti-STIM2 antibody, as indicated. Blots were reprobed with anti-β-actin antibody for protein loading control. Bar graph represents Orai2 (A), Orai3 (B), STIM1 (C) and STIM2 (D) protein expression presented as mean ± SEM of 4 separate experiments. Data were statistically analyzed using Mann-Whitney U-test. **** *p* < 0.0001

Supplementary Fig. 3


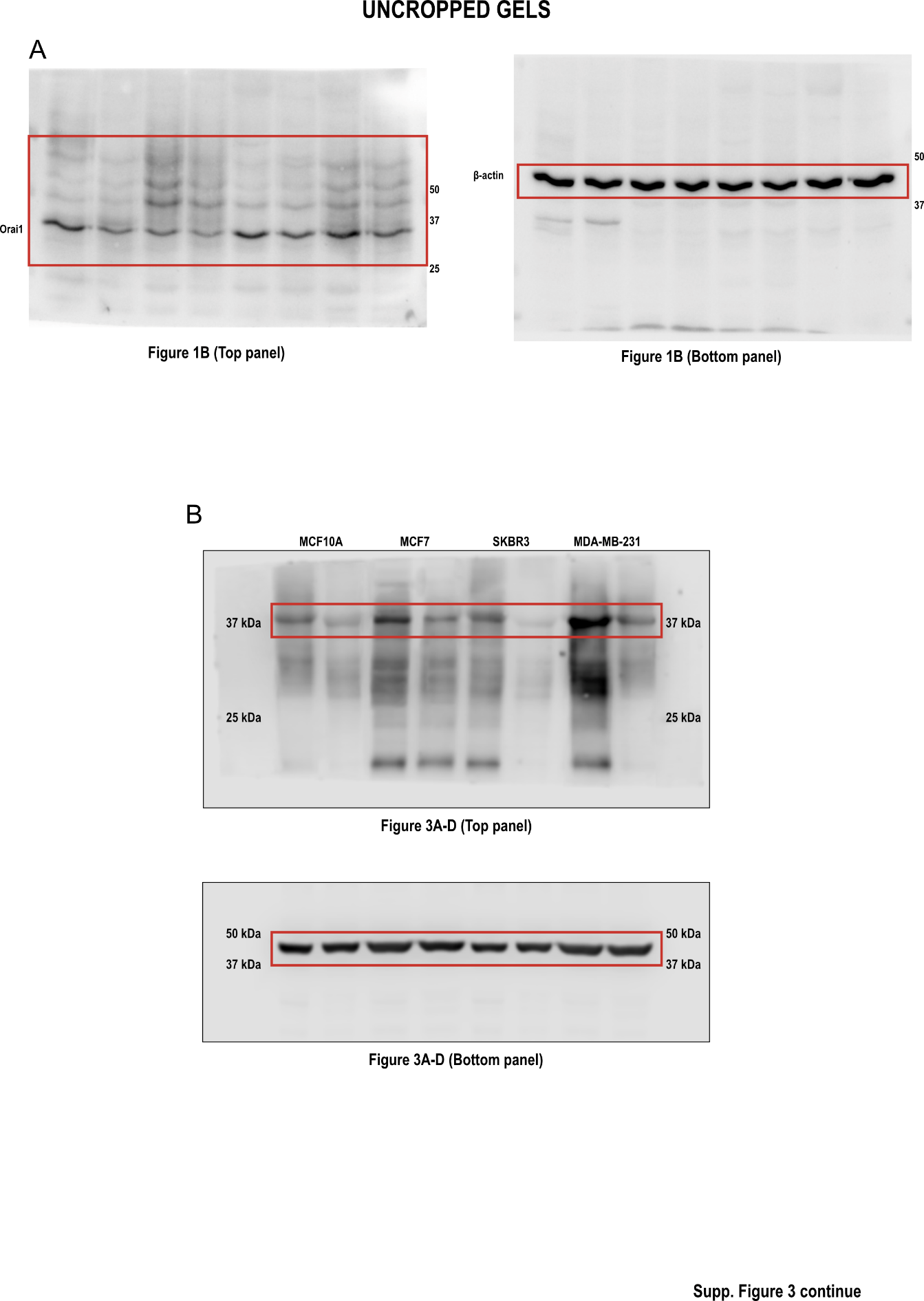


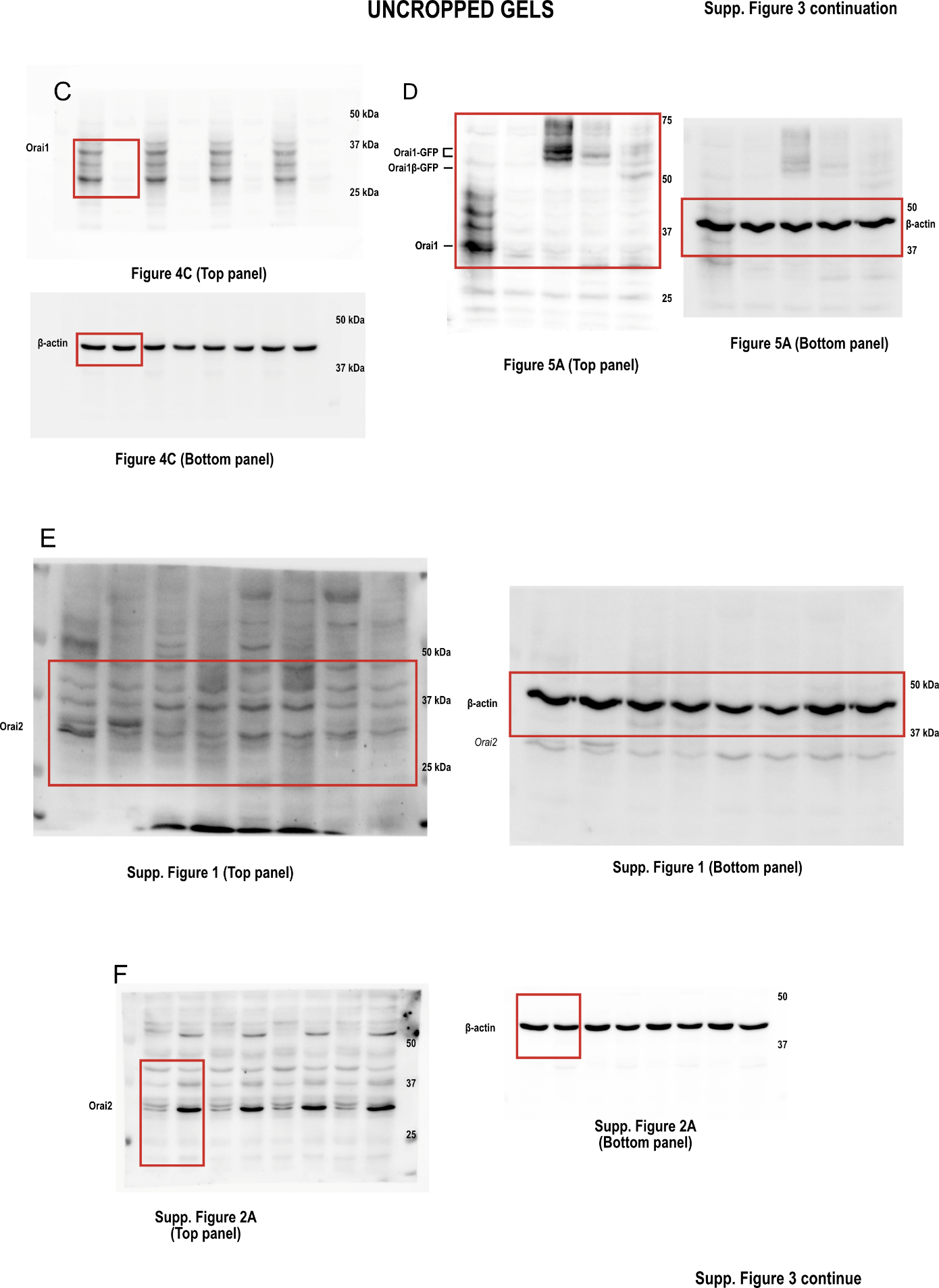


*
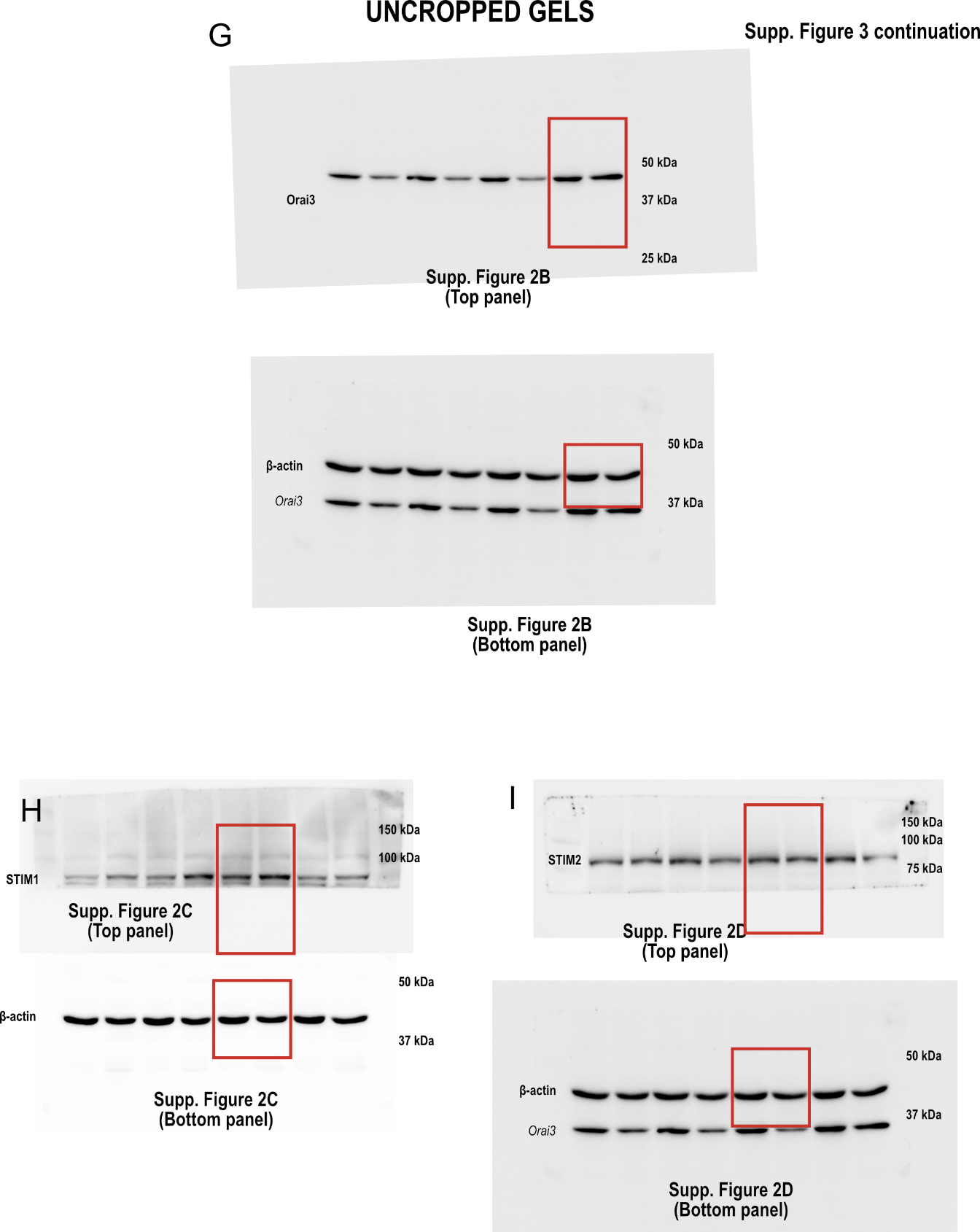
*

**Supplementary Fig. 3.** Original blots used in the manuscript. Uncropped and un-processed blots shown in figure 1B (A), Figure 3A-D (B), Figure 4C (C), Figure 5A (D), supplementary Figure 1 (E), supplementary Figure 2A (F), supplementary Figure 2B (G), supplementary Figure 2C (H) and supplementary Figure 2D (I). Routinely membranes were cut at approximately 65-70 kDa to analyze proteins in the upper and lower membranes simultaneously.
